# Strategies for Empowering activities in Everyday life (SEE 1.0): study protocol for a feasibility study of an Internet-based occupational therapy intervention for people with stroke

**DOI:** 10.1186/s40814-021-00924-x

**Published:** 2021-10-16

**Authors:** Maria Larsson-Lund, Eva Månsson Lexell, Anneli Nyman

**Affiliations:** 1 Department of Health, Education and Technology, Luleå University of Technology, 971 87 Luleå, Sweden; 2 Department of Health Sciences, Lund University, Lund, Sweden; 3 Department of Neurology and Rehabilitation Medicine, Skåne University Hospital, Lund, Malmö, Sweden

**Keywords:** Stroke, Daily activities, Self-management, Lifestyle intervention, Internet-based rehabilitation, Tele-rehabilitation, Digital e-health solutions, Occupational therapy

## Abstract

**Background:**

Rehabilitation after stroke seldom focuses on needs related to an active everyday life and the process of change that people must undergo to adapt to an altered capacity and life situation. In particular, occupational therapy in the late phase needs to support clients in adopting sustainable self-initiated management strategies to regain daily activities and an active everyday life. To improve access to rehabilitation, the use of digital solutions has been suggested. *This study aims* to evaluate the feasibility of the Internet-based occupational therapy intervention “Strategies for Empowering activities in Everyday life” (SEE, version 1.0). We will investigate the feasibility of the intervention process in terms of acceptability and adherence as well as the most suitable outcome measures to evaluate SEE and improve the knowledge about the potential changes and outcomes of SEE for clients with stroke.

**Methods:**

This feasibility study is based on a pretest posttest design without a control group. Quantitative and qualitative data will be collected from clients and staff concurrently embedded in a mixed-method design during the entire study.

**Discussion:**

The project is a first test of a novel Internet-based occupational therapy intervention, and the research will contribute to the continued development and evaluation of the SEE programme. SEE can provide people with strategies in daily activities that can support them to live an active everyday life despite changed capacity and to improve access to rehabilitation interventions.

**Trial registration:**

NCT04588116. *Name of the registry:* Strategies Empowering Activities in Everyday Life (SEE 1.0). A Web-based Occupational Therapy Intervention. *URL of trial registry record*. *Date of registry: Trial first posted*: October 19, 2020; *first submitted*: October 2, 2020

## Background

For people with disabilities, having the right to engage in daily activities and to live an active life is an important issue [1]. People with stroke represent a large group of individuals with disabilities, and yet many face extensive challenges to engage in daily activities. In an investigated sample of people with stroke, more than 50% lacked meaningful daily activities [2]. In particular, this concerns daily activities in society, in places outside the home as well as being able to engage in activities together with other people in their social network [3–8]. Engagement in daily activities includes social, cultural, leisure and domestic activities, e.g. visiting friends, shopping, sports/training and recreation in various forms, commonly limited after stroke [3–8]. Many values attached to daily activities are diminished or lost for people with stroke [8–10] and they are at risk of becoming inactive [11] or having too few activities with little variation of activities during a day [5, 8]. This may affect their ability to achieve a healthy pattern of daily activities [5, 8, 12]. An altered pattern of daily activities, and how people occupy time and space, can disrupt the experience of occupational balance, i.e. the subjective notion of having the right amount of daily activities and the right variation between them [13, 14]. In turn, this can also have a negative impact on their mood and life satisfaction [10, 12, 15–18]. Taken together, regaining daily activities requires interventions that not only restore impairments but also, to a greater extent, focus on the complex needs people face when trying to recreate an active everyday life that is sustainable over time [12, 18–21]. Consequently, rehabilitation interventions also need to address the process of change people must undergo to recreate an active everyday life on new terms [20].

An active everyday life includes engagement in a variety of daily activities at different places within their community, with different people in their social network [22]. The range of complexity is needed to obtain a balance among one’s daily activities. To achieve a healthy pattern of daily activities that facilitate occupational balance, people need to learn to self-reflect and adopt strategies they can use to manage this complexity [5, 12, 22–24]. It is also important to utilize and promote the individual’s ability to mobilize self-initiated management strategies in daily activities during occupational therapy interventions, to overcome or prevent problems in everyday life [5, 8, 23, 25–27].

To improve access to rehabilitation and facilitate tailored interventions to individual needs, the use of E-health solutions has been suggested [28–31]. However, recent research has shown that rehabilitation delivered as E-health interventions are only used to a limited extent [32–35] and often lack a specific focus on promoting an active everyday life. Therefore, we set out to develop an occupational therapy intervention, delivered as an Internet-based programme. The programme “Strategies for Empowering activities in Everyday life” (SEE, version 1.0) aims to support participants in developing management strategies that empower engagement in various daily activities, at different places and together with other people. It will be provided to clients with stroke in the late phase, when access to interventions focusing on empowering an active everyday life is lacking [20, 36–38] and in a phase [20] when people are expected to be ready for change, that is, when they have returned home, and face demands in everyday life.

Consistent with guidelines [39–43] on how complex interventions are developed, several uncertainties must be initially addressed in a feasibility study before moving to larger scale studies. The *clinical uncertainties* are related to the novelty of SEE, in using an Internet-based intervention after stroke [32, 34, 35] and of focusing on self-reflection and self-initiated management strategies for engagement in daily activities. In addition, it also involves as to what changes can be identified in clients after having completed the intervention. Thus, as SEE is an innovative approach, it is important to investigate its acceptability, adherence and value from the perspectives of clients and staff as this may inform potential modifications in the next version of SEE. *Methodological uncertainties* relate to research design concerns, whether the selected assessment tools are appropriate to guide the intervention and evaluate the outcome as well as the variability in outcomes and the relationship to client characteristics. Such knowledge, about the outcome of SEE for different groups, is important when future research design is planned and implemented. These clinical and methodological uncertainties must be addressed in a feasibility study.

### Objectives

The overall aim is to evaluate the feasibility of the Internet-based occupational therapy intervention programme SEE. Specifically, this feasibility study aims to:

- Determine the acceptability, adherence and value of SEE for clients and staff involved in implementing SEE
- Determine the outcome measures and assessment tools to identify changes in clients’ everyday life at 4 and 12 months after completing SEE
- Determine the variability in the potential outcomes of SEE for the clients, and establish the relationship between the clients’ characteristics and scores at onset of SEE.
- Determine the changes experienced by clients in everyday life after SEE and their perceptions of the values of participating in the SEE.
- Determine clients’ experiences of adopting self-management strategies during the intervention process of SEE and determine what facilitates or hinders their adoption of strategies?

## Methods/design

### Study design

This feasibility study is based on a pretest posttest design without a control group to evaluate aspects of feasibility and potential results of the SEE programme. The data collection comprises outcome measurements/assessment tools, qualitative interviews, focus groups and descriptive quantitative data from feasibility registration forms. Consequently, quantitative and qualitative data will be collected concurrently embedded in a mixed-method design of the entire study.

This study, including the SEE programme, follows the Medical Research Council (MRC) guidelines [40, 44] for how complex interventions are developed and evaluated through research in four phases. The present study of the SEE programme involves the second phase of research developing complex interventions, and the design is based on guidance in the area [41, 42, 45]. The study protocol for this feasibility study follows “the Standard protocol items: recommendations for intervention trials 2013 statement” (SPIRIT) [46, 47]. The description of the intervention follows the “template for intervention description and replication” (TIDieR) [48]. The study is also based on guidelines for process evaluation [44], identifying aspects important for sustainability of the implementation in relation to different contexts.

### Study setting

The intervention is performed from outpatient rehabilitation clinics in northern Sweden. The SEE programme is provided through the “support and treatment platform”, an E-health service within the Swedish Healthcare Guide; 1177.se. The client with stroke will perform the Internet-based intervention in their own home or another place of their choice, and the occupational therapist will deliver the intervention from the clinic.

### Eligibility criteria for client participants

Clients with stroke will be included if they fulfil the following inclusion criteria: (a) aged 18–75 years; (b) ≥ 6 months after the onset of the stroke; (c) moderate disability or good recovery after the stroke; (d) access to a screen/computer, Internet and e-ID as well as ability to use them; (e) experience limitations in engagement in daily activities and be motivated to participate in the programme, including being ready for a process of change; and (f) discharged from rehabilitation at hospital or day care. The exclusion criteria are depression, other conditions or diseases that impact daily activities and impairments or other diagnoses that may affect the ability to consent to participation as well as participate in the data collection and intervention. The time frame of 6 months was chosen based on the expectation that clients’ physical recovery has reached a plateau and yet clients still face difficulties in everyday life that has become evident after engaging in daily activities in their own environment for a while with limited access to rehabilitation [49–51].

### Intervention

#### The foundations of SEE 1.0

Comprehensive development approaches inspired the iterative process of designing the intervention programme, involving several steps [52, 53]. The SEE intervention is based on a review of evidence from empirical research and theory. Additionally, SEE was developed and designed based on the involvement of clients and staff in several ways during an iterative process—discussion groups [54], consultations and a first test of the programme at a rehabilitation clinic (the development process, choices, actions and results is reported elsewhere [55]). The programme theory is founded on empirical studies, describing consequences in everyday life after stroke, e.g. [3–8, 12, 27, 56–58], occupational therapy theories about peoples’ daily activities [14, 59–61], person-centeredness [62–65], self-management [33, 49–51, 66–69] motivation [70–72], rehabilitation methodology [73], pedagogical principles of flipped classroom [74–76] and evidence of Internet-based interventions [77–83]. The contribution of these referred components to the programme theory is illustrated in more detail in Table 1.

**Table 1:** An overview of how empirical research and theory have informed the development of the Internet-based occupational therapy intervention SEE

Program theory, components	The contribution to the design of the client intervention	The contribution to the design of the occupational therapist intervention guide and educational program
Evidence of engagement in activities of everyday life, empirical research and reviews	Informed the need and focus of the intervention. Informed the choice of the assessment tools. Informed the content of modules and examples of common changes in engagement in activities of everyday life and self-initiated management strategies in activities.	Guide the activity-based and person-centred reasoning by - Enhancing the understanding of changes in engagement of daily activities and of self-initiated management strategies. - Providing examples from others in a similar situation that can be used to facilitate clients’ change process.
Occupational therapy theory	Informed the design of the intervention on how to focus on a complexity of engagement in activities of everyday life and how to support self-reflection and changes of daily activities as means and ends. Informed the choice of assessment tools. Informed the themes of the modules (see Table 2).	Guide the activity-based person-centered reasoning focusing on: - The complexity of daily activities in relation to health. - Patterns of daily activities. - Occupational balance. - Engagement in activities with different values, at different places and together with others. - How situations can vary when engaging in daily activities, and how clients can be prepared to handle such situation. - Supporting self-reflection of daily activities - Supporting changes of daily activities as means and ends.
Perspectives and principles on person-centeredness	Informed the design to support clients’ reflection on their unique situation and needs in activities of everyday life during the change process.	Guide the person-centred reasoning to focus on the person’s unique needs and situation, with participation, sharing and transparency as important elements. Also, enhancing the reasoning in facilitating clients’ self-initiated management strategies.
Perspectives and evidence of self-management	Informed the design of the intervention process to support the clients to taken on an active role, to identify needs, to adopt strategies and to make an activity plan to act upon in everyday life.	Guide the reasoning about self-management and how to guide the clients to take on an active role in adopting strategies.
Perspectives on motivation	Informed the design on how to support clients to identify their motivation and readiness of change.	Guide reasoning in considering clients’ level of motivation to identify readiness for change and, also, support motivation during the change process. Guide the process and the dialogue with the client by using motivational interviewing to support motivation and facilitate that the client takes on an active role in the change process. Guide how to use questions and how to listen actively to support motivation and autonomy.
Rehabilitation methodology	Informed the process of establishing a plan for a change process.	Guide reasoning in how to actively involve clients in establishing a realistic and feasible activity plan for their change process.
Pedagogical principles and evidence of Flipped-classroom	Informed the design on the digital distance-learning format of the intervention, including short video clips with new knowledge and assignments that are designed to enhance clients’ active role and self-regulated learning.	Guide the dialog with the clients to process content by reflections and discussion to support the clients’ self-reflection to “see” their daily activities in a new way to form a base for the change process.
Perspectives and evidence of Internet-based interventions	Informed the design about potential target groups’ ability to use technology. Informed the design of web-format and the interaction between clients and occupational therapists to enhance feasibility, acceptability adherence and effectiveness	Guide reasoning in clients’ access and ability to use technology, i.e. suitable for this form of intervention. Guide frequencies and types of feedback/ dialogue with clients during the modules to support the change process.

#### Intentions, duration and specific content of SEE 1.0

The SEE facilitates a balanced level of engagement in various daily activities, at different places and together with others supporting an active everyday life. The intention with SEE, which is delivered thoroughly through the Internet, is to support the person to “see” their daily activities in a new light. That is, self-reflect to become aware of their pattern and balance of daily activities, the activities they want to engage in and situations they experience problematic and challenging [14, 59–61]. The purpose of the intervention is also, based on this self-reflection, to support the use of self-initiated management strategies in daily activities [27] that empower the person to take an active role to prevent and overcome problems and challenges in everyday life, in a way that is sustainable over time. For example, strategies to plan and prioritise daily activities over time, choose when and where to engage in an activity, doing preparations, take on action/step at a time, take breaks and ask for help. By empowering people to systematically self-reflect and pay attention to self-initiated strategies in activities so that those that work well are used, people can be supported to take an active role in managing their everyday lives, during their process of change.

The core assumptions of SEE are based on occupational therapy [14, 59–61]. That is, meaningful daily activities are essential for experiences of health and being able to engage in such activities can be complex and influenced by many factors that varies with the situation. The occupational therapy literature also demonstrate how self-reflection and changes can be achieved with daily activities both as a mean and end in the intervention processes. The foundation of the programme is that the person, through self-management [67, 68] and self-initiated management strategies in daily activities [27], can influence experiences of health and consequences of disease or injury by changing their engagement in daily activities. The focus of the programme is that health can be facilitated by the daily activities a person does, where and how and with whom they are performed, their meaning and value and how they are organised in time and in relation to each other [14, 59]. In this sense, health can be achieved despite diseases or injuries.

The person-centred intervention process [62–65] starts with supporting the client to begin a self-reflection process regarding their engagement in daily activities and which self-initiated management strategies they use in activities, which is further addressed in the change process during the intervention. In this, outcome/assessment tools that evaluate the complexity (of the pattern) of daily activities in everyday life and related changes in engagement [14, 59–61] is used. After the self-reflection and evaluation, the intervention continues with an Internet-based programme that is delivered on a secure national health platform that requires a personal e-ID. The program format is built on principles [74, 75] that facilitate the clients’ learning and responsibly in working with changes. The programme comprises eight modules: one introduction module focusing on needs and motivation for change and seven modules focusing on engagement in daily activities and self-initiated management strategies that support development of an active everyday life (Table 2). In a flipped classroom manner [74, 75], each module includes a short educational video lasting 5–10 min, followed by digital assignments supporting the person to become aware of their engagement in daily activities in a new way and to identify new management strategies. In accordance with the intervention guide, the occupational therapist provides person-centred feedback after each assignment and provides access to the next module. Furthermore, the client and occupational therapist meet three times for online face-to face guiding sessions. This part of the programme is expected to take 2–3 weeks. Thereafter, the client and OT collaborate [73] to establish an activity plan to further support clients in defining, prioritising and achieving their prioritised person-centred goals. The long-term goal in “the activity plan for an active everyday life” is to facilitate a healthy and balanced pattern of daily activities, while the short-term goals focus on specific daily activities and the development of self-initiated management strategies supporting desired changes involving daily activities, places and other people. During the change process, the person receives individually tailored support from the OT, and the intervention continues until the goals are achieved. Depending on individual goals in the activity plan and subsequent strategies/actions, sessions may be provided at the client’s home or other places important for their daily activities outside the home or visits at the clinic, in addition to continued online sessions supporting the progression of the change process provided for all clients.

**Table 2:** Overview of the Internet-based occupational therapy intervention “Strategies for empowering activities in everyday life” (SEE)

**Module theme**	**Content**	**Assignment**	**Client activity**	**Occupational therapy (OT) guidance**
Introduction module	The foundation, aim, design of the web-programme, modules and content.		Listen to the module presentation	
1. Changes in daily activities and participation after stroke	Consequences of a stroke in daily activities, places and social networks	Reflect on whether you recognise these changes in your life situation and if you are interested in participating in the programme	Send in a short note	Contact the client for a meeting and dialogue of how to move forward with the programme
2. Value and meaning of daily activities	How daily activities generate different values and contribute to meaning in life	Reflect on which activities bring meaning and health in your life.	Send in 3–5 important activities in your everyday life before the dialogue with the OT after module 4	Provide confirming, reflecting and encouraging feedback
3. Activity pattern	How activity patterns, time use, roles, habits, routines, time for recovery and rest influence health	Reflect on your variation in daily activities, places for activities, activities alone and with others, and time use based on your activity diary (completed during the initial assessment)	Complete a form about your satisfaction with time spent on various daily activities before the dialogue with OT after module 4	Provide confirming, reflective and encouraging feedback
4. Complexity of daily activities	How different daily activities—main activities, preparatory activities and unexpected events—create patterns and how they can disturb the activity	Reflect on your daily activities and how they have changed, what unexpected events disturbs your activities	Make a short note	Contact the client for a meeting on the reflections made in the assignments
5. Occupational balance	How occupational balance is influenced by the activity pattern. How one’s activity pattern is connected to other peoples’ patterns. The importance of involving others in the change process.	Reflect on your occupational balance between different activities during a week	Complete a form and make a short note about desired changes and challenges for dialog with OT after module 7	Provide confirming, reflective and encouraging feedback
6. Strategies that support daily activities	How strategies for daily activities, places and social networks support management and control over everyday life	Reflect on strategies that you use at present to support engagement in daily activities	Complete a form for strategies used presently, make a short note of strategies supporting your daily activities for dialogue with OT after module 7	Provide confirming, reflective and encouraging feedback
7. Prioritise and make changes in daily activities to support an active life	The change process, prioritising desired activity pattern and daily activities here in, identifying strategies supporting change	Reflect on how you can initiate and achieve change in everyday life. What do you prioritise, and which strategies are needed to achieve desired changes?	After taking module 7, mark that you are finished.	Contact the client for a meeting and dialogue on the reflections made in the assignments. Agree on an intervention plan, including long-term and short-term goals, to regain and make changes in activity patterns and daily activities supporting an active life. Rehabilitation will continue until goals are reached.

#### Education programme and supervision of the occupational therapists (OTs) in the intervention delivery of the SEE 1.0

The purpose of the education programme is to ensure that the SEE is provided as intended in a uniform and standardised way by the OTs. The education programme is based on a comprehensive intervention guide and several recorded videos, covering the foundation of SEE: (a) background of the need for new Internet-based solutions in the late phase of rehabilitation; (b) summary of empirical findings of changes in engagement in daily activities in persons with stroke, e.g. [3–5, 8, 10, 12]; (c) summary of occupational therapy theories about human daily activities [14, 59–61]; (d) person-centeredness [62–65], self-management [67, 68] and flipped classrooms [74]; (e) motivating interviewing [72]; and (f) management strategies in daily activities [27]. The intervention guide includes detailed information about programme goals, the different modules and the procedures the OTs are expected to undertake to guide their clients after each module.

The education programme is delivered in a flipped-classroom manner [74]. In addition to the educational videos, it includes information about the material for self-tutorials (including the intervention guide) with subsequent workshops to clarify issues related to the implementation and delivery of the programme in the platform and through online sessions. Additionally, during the intervention period, the OTs will receive supervision from the research group to clarify questions that arise related to the implementation of the intervention.

### Participant timeline

Enrolment of the 30–40 client participants begun in November 2020 and is expected to be completed by June 2022. Due to the person-centred format of the programme, involving a change process, the length of the intervention process with the OTs is expected to vary between 3 and 5 months. However, the participants’ change process is expected to continue up to 1 year after the intervention started [84], which explains why outcome measures will be conducted up to 12 months after starting the intervention.

The outcome measures and sociodemographic data will be conducted at baseline before the programme is initiated (0 months), and outcome measures will be further conducted at 4 and 12 months after the intervention starts. The feasibility registration forms and logbooks will be completed by the OTs for each client participant after each session throughout the intervention process. The study-specific feasibility questionnaires will be collected from the client participants after 4 and 12 months. The individual interviews with the clients will be conducted 1 and 4 months after entering the SEE. Focus group interviews with OTs will be performed on three to four occasions scheduled at random times during the study period. The focus groups with the management and other keypersons/stakeholders will occur one to two times after the intervention has been completed.

### Sample size

As this is a feasibility study, no power calculation is required [41, 85]. The sample size justification is instead based on the premises that the sample has to be sufficiently large to provide enough detail and information on the intervention. That is, on the aspects of feasibility investigated and potential outcomes [41]. It is important that the sample is representative of the target population. Additionally, as the OTs will become experienced in applying the new programme and providing Internet-based interventions the required sample size is estimated to be 30–40 clients with stroke.

### Recruitment procedures, including informed consent

The screening of potential participants will be conducted in two steps: first based on the information in the potential participant’s register and second on evaluation of their current status. Initially, staff at the rehabilitation clinics will identify potential clients based on the inclusion (diagnosis, age, time since illness, ability to express themselves orally and in writing) and exclusion criteria (other health conditions). Potential clients are sent an information letter and asked to respond whether they are interested to participate. If responses are not received within 1–2 weeks, the potential participants will be contacted by telephone to ensure that no volunteers are missed. The potential clients who show interest in participating will provide consent for their contact information to be transferred from the clinic to the researchers. Thereafter, the researchers will contact the participants by telephone and repeat the information presented in the information letter orally and respond to any questions. The participants are also informed about possible risks and benefits of participation (as stated in ethical considerations) and about the right to withdraw at any time without explanation. If written consent is given, the participants will be further screened for eligibility criteria, and the second screening step will be booked as an e-meeting (video call) to finally determine whether the participant is representative of the target population. During the screening, assessment tools will be used to evaluate whether the participants meet the criteria for the severity of disability [86], have access to a screen/computer, Internet and e-ID and can use them, not have an ongoing depression [87], experience limitation of engagement in daily activities and are motivated to undergo a change process at that time [70, 71].

The recruitment of the OTs will be based on those who are involved with the clients. Additionally, different managers and other keypersons/stakeholders in organisations related to health care and social services will be included in a “reference panel” of 5–8 persons. The staff will receive an information letter about the study with an opportunity to give their written informed consent to participate or decline without explanation.

### Outcomes and data collection methods

#### Feasibility of the intervention

Study-specific feasibility registration forms and intervention logbooks will be used to collect data about the implementation of the programme (feasibility: acceptability, adherence and value). These will be completed by the OTs after each client session during the entire intervention process.

The feasibility registration forms contain questions about delivery, compliance, dose and reach of the intervention to detect deviation in relation to the intervention guide for each client. In the intervention log books, field notes will be recorded regularly about how each client manages to use the Internet programme and implement strategies for change. Furthermore, the notes will include how the (online) therapeutic relationship and communication are working as well as unintended harms. OTs will also make notes about their own experiences of using SEE. These data will be complemented by focus group interviews with the OTs who provide the intervention. The interviews will cover experiences with SEE, such as the mode of delivery, dose, content and logic order of the modules, barriers and therapeutic components, and whether expected changes occur or not. The interviews will also cover how thoroughly the intervention guide was followed and whether the education received prior to starting the programme prepared them sufficiently to support the clients in their change processes.

A study-specific questionnaire concerning the acceptability and value of SEE from the clients’ perspective will also be collected. The questions will cover experiences of the satisfaction with intervention, such as the mode of delivery, dose, content of the modules, therapeutic relationship and communication online, as well as unintended harms and the extent to which the intended outcomes/benefits of the SEE are achieved. The clients self-report their satisfaction of the various aspects of the intervention on a 4-point ordinal scale ranging from not at all satisfied to very satisfied and the benefits of the intervention on 4-point ordinal scale ranging from do not agree at all to strongly agree.

The reference panel will take part in focus groups to identify whether SEE can be transferred and implemented in clinical practice and clinics in other contexts. This data collection will be based on process evaluation [44] that can be conducted in parallel with the intervention to investigate implementation, the role of context and mechanisms of impact.

#### Experienced changes

To determine the experiences of the intervention, qualitative research interviews [88] will be performed with a purposeful selection of the client participants. The purposive selection process [89] will continue until a variety of experiences occurs, ensuring that richness in data is achieved. The interviews will be semi-structured and conducted on two occasions during the intervention process. The questions capture experiences of the intervention/rehabilitation process and how the process influences everyday life. Questions will specifically focus on how they adopt self-initiated management strategies to overcome or prevent challenges in their engagement in daily activities, possibilities and hindrances in the process of recreating an active everyday life. The initial interview also explores the experiences of changes in engagement in daily activities and management strategies used before participating in the intervention as well as potential new insights into needed changes after the first weeks in the intervention. During the interviews, techniques that enhance the validity of interviews of people with cognitive impairments will be used [90]. The interviews will be recorded and transcribed verbatim transcribed verbatim without violating confidentiality.

#### Potential SEE outcomes

The potential outcomes of SEE will be determined using the following standardised assessment tools; The Profiles of Occupational Engagement [91] is based on an interview of a 24-h completed diary of time use. The nine items are scored on an ordinal scale and a higher score indicate a higher level of engagement in daily activities. The Occupational Balance Questionnaire (OBQ) [92] consist of 11 items that are summed to a total score, ranging between 0 and 33. A higher score implies more satisfaction with the amount and variation of occupations, i.e., a higher level of occupational balance. The Occupational Value predefined (Oval-Pd) [93] includes18 items that are summed into a general occupational value, ranging between 18 and 72. A higher score indicates that the respondent is frequently engaged in valued daily activities. The Life Satisfaction Questionnaire (Lisat-11) [94, 95] consist of 11 items, including self-reports of satisfaction with life as a whole and physical and mental health. The items are rated on ordinal scale ranging from 1 to 6 and a higher score reflect a higher level of satisfaction. How self-management is realised will be assessed using the General Self-Efficacy Scale (GSE) [96, 97] that includes 10 items that are summarized to a score ranging between 10 and 40. A higher score reflects greater sense of general self-efficacy. Actual and perceived work ability will be evaluated for those of working age. The Work Ability Index (WAI) [98] examines self-perceived work ability on a 10-point ordinal scale. A higher score reflects a higher level of perceived work ability. Goal achievement in the activity plan will be noted by the OTs.

Sociodemographic data will be collected using a registration form. To evaluate aspects of the medical disorder, the Glasgow Outcome Scale Extended (GOSE) [86], Mental Fatigue Scale (MFS) [99] and Hospital Anxiety and Depression Scale (HAD) [87] will be used at baseline.

### Data management

All the data will be transferred by the research group to a word processing or calculation computer programme and securely saved anonymously at the data storage service of Luleå University of Technology, Sweden, according to the rules and guidelines for research ethics [100, 101] and the GDPR [102]. The names, contact information and consent forms will be stored on paper, together with their identification (ID code) number, in a cabinet separate from the data to make it impossible to identify persons*.*

### Analysis

#### Feasibility of the intervention

The feasibility registration forms and study-specific questionnaires will be analysed using descriptive statistics and quantitative content analysis [103] to outline the delivery, compliance, dose, reach of the intervention, adherence and values. The intervention log books, including field notes and transcribed focus group interviews with OTs, will be analysed using qualitative content analysis [104] and the acceptability, adherence and values of SEE will be described. Additionally, the clients’ experiences of acceptability and adherence will be analysed using qualitative content analysis. The data from the focus groups with the reference panel of managers and key stakeholders will be analysed to reveal the contents and meanings inherent in the discussions of acceptability in a wider context [105]. To identify whether the assessment tools are appropriate to measure changes that may occur after SEE, the match between the experienced values and meanings of the intervention from the clients’ perspective (study-specific feasibility questionnaires, registration forms) and the outcomes identified by the assessment tools used will be compared descriptively by content analysis.

#### Experienced changes

The interview transcripts of the clients’ experiences of the intervention process in SEE will be analysed using grounded theory [106] to uncover the characteristics and meanings of interactions in the process.

#### Potential SEE outcomes

Patterns of changes within the group will be determined by descriptive statistics, comparison and non-parametric tests on all the outcome measures (POES, OBQ, Oval- PD, LiSat-11, GSE, WAI) and, also, by goal achievement. The data will be analysed over time (from baseline to 4 months and 12 months). To determine the variability between the outcomes (at 4 months and 12 months) and background data, logistic regression will be used for dichotomous outcomes and linear regression will be used for continuous outcomes. The statistical calculations will be performed with SPSS version 27.0 [107] and the *p*-value will be set to *p* <0.05 for all statistical tests.

### Ethics and dissemination

The project will be conducted in agreement with research ethics [100, 101] and the GDPR [102]. The reference panel will be involved in how and where to disseminate the results to patients and the public through publications in peer-reviewed scientific journals, websites and presentations at conferences.

## Discussion

This study will provide important knowledge about SEE that can add to the continued development of the programme. If the results confirm intervention feasibility and show that the potential outcomes contribute to positive changes of daily activities for clients with stroke, the intention is to continue to refine and evaluate the intervention. This evaluation will be performed first in a pilot RCT study and, later, in large-scale studies. Our longer-term goal is also to adapt and test SEE in other groups (for instance, people with other neurological disorders and older people), to develop other delivery forms (group-based intervention), including merging the SEE into a multi-disciplinary rehabilitation program. Otherwise, if changes that are more extensive are needed, the programme will be further developed to better suit clients, OTs and clinical practice.

Because the intervention is new, several uncertainties exist that are common when developing and evaluating interventions. To address these issues, the design involves a combination of methods, capturing quantitative and qualitative data, which will complement each other to provide a more complete picture of the strengths and weaknesses of the programme. Patient and public involvement is crucial in developing new interventions and disseminating new knowledge. Thus, as reflected above, the present study is designed to improve SEE by the feedback provided by clients, OTs and the reference panel. The intervention is unique because it combines an Internet-based format with a face-to-face meeting online, and the content focusing on empowering self-initiated strategies for an active everyday life is innovative and not part of clinical practice today. To address the complexity between engagement in daily activities, an active life and health, many aspects must be considered. This notion is reflected in the content of the intervention, with a focus not only on the actual performance in a specific daily activity but also on patterns of daily activities, time use, level of engagement and routines. Furthermore, subjective experiences related to engagement include value, meaning, satisfaction and occupational balance. Consequently, having an active everyday life, reflecting a healthy pattern of daily activities and a satisfying (balanced) level of engagement in activities, is not so easily self-managed by people. SEE has the potential to provide people with tools, in the form of self-reflection and self-initiated management strategies in daily activities that can support them sustainably to live an active everyday life, even if individual capacity or environmental aspects change over time. The results of this study can also inform future Internet-based occupational therapy interventions and the clinical research of people with needs related to an active everyday life.

## Data Availability

The datasets collected and analysed during the current project are available from the corresponding author on reasonable request.
